# Prevalence and Profiles of Risky Driving Behavior Among US Teenagers

**DOI:** 10.1001/jamanetworkopen.2024.25263

**Published:** 2024-07-31

**Authors:** Johnathon P. Ehsani, Michelle L. Duren, Brydon J. B. Grant, Ahmed Sabit, Gayane Yenokyan

**Affiliations:** 1 Department of Health Policy and Management, Johns Hopkins Bloomberg School of Public Health, Baltimore, Maryland; 2 Department of Epidemiology and Environmental Medicine, The University at Buffalo, Buffalo, New York; 3 Baháʼí Institute for Higher Education, Iran; 4 Department of Biostatistics, Johns Hopkins Biostatistics Center, Baltimore, Maryland; 5 Johns Hopkins University Bloomberg School of Public Health, Baltimore, Maryland; 6 Department of Biostatistics, Johns Hopkins Biostatistics Center, Baltimore, Maryland; 7 Johns Hopkins University Bloomberg School of Public Health, Baltimore, Maryland

## Abstract

This survey study updates prevalence data on risky driving behaviors in a nationally representative sample of young people in the US and characterizes profiles of drivers according to the types of risky behaviors they engaged in.

## Introduction

The decade-long downward trend in crash deaths among teenagers was reversed during the COVID-19 pandemic. In 2021, 2116 young drivers died in traffic crashes, an 11% increase from 2020.^1^ The Centers for Disease Control and Prevention’s latest data on risky driving behaviors among young people were compiled in 2021.^2^ The purpose of this study was to update prevalence data on risky driving behaviors in a nationally representative sample of young people in the US and characterize profiles of drivers according to the types of risky behaviors they engaged in.

## Methods

We conducted a nationally representative survey study of teenagers (aged 16-19 years) between May 4 and June 10, 2022. Participants were recruited from NORC’s AmeriSpeak platform, a probability-based panel of randomly sampled US households.^3^ Informed consent was provided by the respondents. The Johns Hopkins Bloomberg School of Public Health institutional review board approved this study. We followed the AAPOR reporting guideline.

Risky driving scales were adapted from the Youth Risk Behavior Survey (YRBS).^2^ Respondents were asked whether they drove in the past 30 days, whether they engaged in a range of risky driving behaviors in the past 30 days, and whether they had been in a crash in the past year.

Prevalence estimates and 95% CIs incorporated sampling weights to generate nationally representative estimates. Latent class analysis was performed to identify subgroups (eMethods in Supplement 1). Odds ratios were calculated using logistic regression models with crash involvement as the outcome. Analyses were conducted using R, version 4.2.2.

## Results

The survey completion rate was 42.8% with a final sample of 267 teenagers aged 16 to 19 years (median age, 17.5 years; range, 16-19 years [IQR, 17-18]; 130 female [48.4%]). Two distinct groups of drivers were identified as high (n = 36) and low (n = 231) risk. The Table presents the prevalence of risky driving behaviors overall and by high- and low-risk groups. Approximately two-thirds of the sample (63.0% [95% CI, 55.3%-70.0%]) reported at least 1 risky driving behavior. Over half the sample (52.8% [95% CI, 44.9%-60.6%]) reported texting or emailing while driving. Over one-fifth reported inconsistent seat-belt use (23.8% [95% CI 17.4%-31.7%]) and driving after using marijuana (20.8% [95% CI, 14.6%-28.7%]). One in 6 teenagers reported driving after drinking alcohol (16.3% [95% CI, 10.9%-23.7%]), and (9.6% [95% CI, 5.4%-14.6%]) reported driving after use of any other drug. Approximately 1 in 6 respondents reported being in 1 or more crashes (15.6% [95% CI, 10.9%-21.8%]) in the past year. The high-risk group accounted for most risky driving behaviors and reported significantly more crashes than the remaining sample (Figure).

**Table.** Overall Prevalence of Risky Driving Behaviors and by Low- and High-Risk Groups

Characteristic	Point estimate, % (95% CI)		
	Overall prevalence	Risk group	
		Low	High
Risky driving behavior in the past 30 d			
≥1 Risk behaviors^a^	63.0 (55.3-70.0)	56.3 (47.9-64.4)	100
Any texting or emailing	52.8 (44.9-60.6)	45.7 (37.2-54.2)	92.6 (78.0-97.8)
Not always wearing seat belt	23.8 (17.4-31.7)	15.3 (9.7-23.4)	70.8 (45.8-87.5)
Any drinking alcohol	16.3 (10.9-23.7)	4.6 (1.7-11.4)	81.8 (54.4-94.2)
Any marijuana use^a^	20.8 (14.6-28.7)	6.6 (3.0-13.9)	100
Any other drug use^a^	9.6 (5.4-14.6)	0.3 (0.1-0.7)	57.1 (34.0-77.5)
≥1 Crashes^a^^,^^b^	15.6 (10.9-21.8)	6.1 (3.5-10.3)	68.7 (42.5-86.7)

^a^ Item is not a question included on the Youth Risk Behavior Survey.

^b^ Item corresponds to whether the teenager had been involved in 1 or more crashes while driving in the past 12 months.

**Figure.**
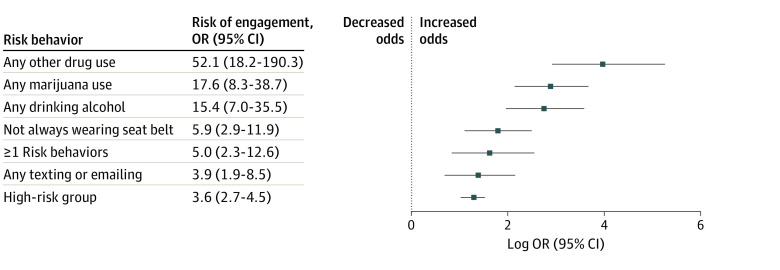
Odds of Crash Involvement Based on Risk Factors OR indicates odds ratio.

## Discussion

The prevalence of risky driving behaviors in our sample provides an update on the most recent data from the 2021 YRBS.^4^ Differences in this sample included a higher prevalence of distracted driving relative to the YRBS (52.8% vs 36.1%) and driving after drinking alcohol (16.3% vs 4.6%), but a lower prevalence of not always wearing a seatbelt (23.8% vs 39.9%). These differences could be because of the older age of this sample. Our survey found that 1 in 5 teenagers reported driving after marijuana use, which is not measured in the YRBS. A minority of young drivers accounted for the most risky behaviors and crashes. Screening and targeted intervention for the highest-risk population could be conducted within the graduated licensing framework using vehicle telematics.^5^

The NORC AmeriSpeak panel used probability-based recruitment consistent with best-practice standards for survey research, but these results may still be vulnerable to sampling biases. Risky driving behaviors could be underreported because of social desirability or recall bias.

## Notes

Supplement 1. **eMethods.** Methods for Latent Class Analysis
eReference.

Supplement 2.
Data Sharing Statement
