# Supplementary material for: Prevalence and Profiles of Risky Driving Behavior Among US Teenagers
Source: JAMA Netw Open. 2024 Jul 31;7(7):e2425263. doi: 10.1001/jamanetworkopen.2024.25263 (PMC11292446; doi:10.1001/jamanetworkopen.2024.25263)
Supplement: Supplement 1. — eMethods. Methods for Latent Class Analysis eReference. [file jamanetwopen-e2425263-s001.pdf]

Supplemental Online Content

Ehsani JP, Duren M, Grant B, Sabit A, Yenokyan G. Prevalence and profiles of risky driving behavior among US teenagers. *JAMA Netw Open*. 2024;7(8):e2425263. doi:10.1001/jamanetworkopen.2024.25263

eMethods. Methods for Latent Class Analysis
eReference.

This supplemental material has been provided by the authors to give readers additional information about their work.

© 2024 Ehsani JP et al. *JAMA Network Open.*

eMethods. Methods for Latent Class Analysis

The polytomous latent class analysis was conducted in R.[1] Initially, individuals are assigned the same probability of being assigned to each of the latent classes. The number of classes is increased progressively. At each step, assignments are adjusted so that the probability of the dichotomous variables of adverse driving behavior that belong to a particular class will have similar characteristics. As a result, the class membership probabilities change. The differences between the adjusted model and the observed data are the errors that decrease as the number of classes is increased. The maximum likelihood is used to estimate the model using the expectation-maximization algorithm. The number of classes was increased from 2 to 9 to verify that a global rather than a local maximum is reached. At each level, the model was replicated at least ten times, the model with the greatest likelihood was selected. The tradeoff in reducing error is offset by increasing the number of classes is optimized objectively by minimizing the Bayesian information criterion. The validity of the final model is judged by determining differences in the crash rates between the different classes in the optimized model.

© 2024 Ehsani JP et al. *JAMA Network Open.*

eReference.

1. Linzer DA, Lewis JB. poLCA: An R package for polytomous variable latent class analysis. *Journal of statistical software*. 2011;42:1-29.

© 2024 Ehsani JP et al. *JAMA Network Open.*
