# Supplementary material for: Prevalence and Profiles of Risky Driving Behavior Among US Teenagers
Source: JAMA Netw Open. 2024 Jul 31;7(7):e2425263. doi: 10.1001/jamanetworkopen.2024.25263 (PMC11292446; doi:10.1001/jamanetworkopen.2024.25263)
Supplement: Supplement 2. — Data Sharing Statement [file jamanetwopen-e2425263-s002.pdf]

Data Sharing Statement

Ehsani. Prevalence and Profiles of Risky Driving Behavior Among US Teenagers. *JAMA Netw Open*. Published August 01, 2024. doi:10.1001/jamanetworkopen.2024.25263

Data
Data available: No

Additional Information
Explanation for why data not available: These data were collected from minors and require IRB approval to be shared with other investigators.
